# Pan-genome analysis of the emerging foodborne pathogen *Cronobacter* spp. suggests a species-level bidirectional divergence driven by niche adaptation

**DOI:** 10.1186/1471-2164-14-366

**Published:** 2013-05-31

**Authors:** Christopher J Grim, Michael L Kotewicz, Karen A Power, Gopal Gopinath, Augusto A Franco, Karen G Jarvis, Qiong Q Yan, Scott A Jackson, Venugopal Sathyamoorthy, Lan Hu, Franco Pagotto, Carol Iversen, Angelika Lehner, Roger Stephan, Séamus Fanning, Ben D Tall

**Affiliations:** 1 CFSAN, FDA, Laurel, USA; 2 Oak Ridge Institute for Science and Education, Oak Ridge, USA; 3 UCD Centre for Food Safety, School of Public Health, Physiotherapy & Population Science, University College, Dublin & WHO Collaborating Centre for Cronobacter, Belfield, Dublin, Ireland; 4 Food Directorate / Direction des aliments, Bureau of Microbial Hazards / Bureau des dangers microbiens, Health Canada / Santé Canada, and Sir F.G. Banting Research Centre / Centre de recherches Sir F.G. Banting, Ottawa, ON, Canada; 5 Nestle Research Center, Lausanne, Switzerland; 6 Institute for Food Safety and Hygiene, University of Zurich, Zurich, Switzerland

## Abstract

**Background:**

Members of the genus *Cronobacter* are causes of rare but severe illness in neonates and preterm infants following the ingestion of contaminated infant formula. Seven species have been described and two of the species genomes were subsequently published. In this study, we performed comparative genomics on eight strains of *Cronobacter*, including six that we sequenced (representing six of the seven species) and two previously published, closed genomes.

**Results:**

We identified and characterized the features associated with the core and pan genome of the genus *Cronobacter* in an attempt to understand the evolution of these bacteria and the genetic content of each species. We identified 84 genomic regions that are present in two or more *Cronobacter* genomes, along with 45 unique genomic regions. Many potentially horizontally transferred genes, such as lysogenic prophages, were also identified. Most notable among these were several type six secretion system gene clusters, transposons that carried tellurium, copper and/or silver resistance genes, and a novel integrative conjugative element.

**Conclusions:**

*Cronobacter* have diverged into two clusters, one consisting of *C*. *dublinensis* and *C*. *muytjensii* (Cdub-Cmuy) and the other comprised of *C. sakazakii, C. malonaticus, C. universalis,* and *C. turicensis*, (Csak-Cmal-Cuni-Ctur) from the most recent common ancestral species. While several genetic determinants for plant-association and human virulence could be found in the core genome of *Cronobacter*, the four Cdub-Cmuy clade genomes contained several accessory genomic regions important for survival in a plant-associated environmental niche, while the Csak-Cmal-Cuni-Ctur clade genomes harbored numerous virulence-related genetic traits.

## Background

*Cronobacter* is a newly described genus that includes opportunistic pathogens formerly classified as *Enterobacter sakazakii*[1]. *E. sakazakii* was first described by Farmer et al. [2], using DNA-DNA hybridization studies and phenotyping to reclassify a group of yellow-pigmented *Enterobacter cloacae* isolates attributed to cases of neonatal meningitis [3] into 15 phenotypically distinct biogroups. A 16^th^ biogroup was later described [4]. Although *E*. *sakazakii* was synonymous with the original single species epithet, Iversen et al. [1], using a polyphasic approach based on extensive genotypic and phenotypic criteria, reclassified strains of this diverse species within the novel genus, *Cronobacter*. Originally, the genus contained six recognized species, *C*. *sakazakii* (Csak, biogroups 1–4, 7, 8, 11, and 13), *C*. *malonaticus* (Cmal, biogroups 5, 9, and 14), *C*. *muytjensii* (Cmuy, biogroup 15), *C*. *turicensis* (Ctur, biogroup 16), *C. dublinensis*, subsp*. dublinensis, lausannensis,* and *lactaridi* (biogroups 12, Cdubdub, 10, Cdublau and 6*,* Cdublact, respectively) and *C*. genomospecies 1. Joseph et al. [5] have updated this taxonomy by designating members assigned to the genomospecies group 1 as *C*. *universalis* (Cuni, biotype 16c) and identifying a new species, *C*. *condimenti*, based on a single known strain using a seven gene multi-locus sequence typing (MLST) scheme.

*Cronobacter* have been primarily associated with infections in infants, but recent reports have highlighted the risk posed to immune compromised adults, particularly the elderly [6,7]. *Cronobacter* have been recovered from a wide variety of foods, including powdered infant formula (PIF), follow-on formulas, weaning foods, milk and sodium caseinate powders, rice seed, dried herbs, teas, and spices, spiced meats, dried flour or meal (corn, soy, potato, wheat, and rice), dried infant and adult cereals, dried vegetables, grains, tofu, powdered ice tea, mixed salad vegetables, tomato harvesting bins, chocolate, candied cough drops, and pastas [8-12]. Nevertheless, PIF remains epidemiologically linked to outbreaks of neonatal and infantile meningitis, and *Cronobacter* are routinely isolated from household PIF preparation equipment, including blenders and spoons. Although PIF is regarded as an important source of this pathogen, the main reservoir for *Cronobacter* seems to be the environment [13].

*Cronobacter* are capable of surviving and persisting in low-water environments [8], owing to their distribution in dry food goods. Although the exact genetic determinant remains to be identified, Breeuwer et al. reported that intracellular accumulation of trehalose, among other features, prevents protein denaturation and membrane fusion and these may contribute to the desiccation-resistant properties of the bacterium [14,15]. The expression of an exopolysaccharide composed of cellulose may also be required [16]. Thermoresistance of *Cronobacter* has also been reported [14,17]. Proteins unique to thermotolerant *Cronobacter* strains have been identified by liquid chromatography and mass spectrometry; for example, a protein, Mfla_1165, which is a homologue of a hypothetical protein from the thermotolerant bacterium *Methylobacillus flagellatus* KT [18]. Recently, an 18-kb region containing 22 open reading frames that are up-regulated under heat adaptation growth conditions has also been reported [19]. The major feature of the region is a cluster of conserved genes, which have significant homology to proteins involved in bacterial stress responses, including heat, oxidation, and acid.

Previously, Kucerova et al. [20] reported on the genome sequence of *C. sakazakii* ATCC BAA-894, which was isolated from a PIF product that was used in a neonatal intensive care unit and which gave rise to an outbreak in 2001 in Tennessee, USA. In an attempt to further determine virulence factors and mechanisms of pathogenicity in this bacterium, the genome of *C. turicensis* z3032, cultured from the blood of one child with meningitis [21] was sequenced [22]. In this paper, we describe the genomes of the type strains of *Cronobacter dublinensis* subsp. *dublinensis*, *Cronobacter dublinensis* subsp. *lactaridi*, *Cronobacter dublinensis* subsp. *lausannensis*, *Cronobacter malonaticus*, *Cronobacter muytjensii*, and *Cronobacter universalis*. Comparative genomics was performed on these six genomes, as well as two previously published, closed genomes of *Cronobacter*. We identified and characterized the features associated with the core and pan genome of this bacterial genus in an attempt to understand the evolution of these organisms and the genetic content of each species.

## Results

### General features of sequenced *Cronobacter* genomes

Whole genome sequencing of six species type strains of *Cronobacter* was performed (Table 1). Each genome analyzed in this study contained a single chromosome and a large repFIB plasmid, similar to Csak BAA-894 (pESA3) and Ctur z30232 (pCTU1), except the genome of *C*. *muytjensii* ATCC 51329. Previously, Franco and Hu et al. [23] reported that only 75% of strains of *C*. *muytjensii* harbored this plasmid, while the incidence of this plasmid in other *Cronobacter* spp. is 99%. The G + C% content of each genome ranged from 55 to 57% (Table 1). Each of the six draft genomes contained seven ribosomal RNA operons, as inferred from comparative genomics with Csak BAA-894 and Ctur z3032 (Table 1).

**Table 1:** General features of genomes used in this study

**Strain**	***C. malonaticus *LMG 23826**	***C. dub. dublinensis *LMG 23823**	***C. dub. lausannensis *LMG 23824**	***C. dub. lactaridi *LMG 23825**	***C. muytjensii *ATCC 51329**	***C. universalis *NCTC 9529**	***C. sakazakii *ATCC BAA-894**^**a**^	***C. turicensis *z3032**^**a**^
Contigs/chromosomes	69	41	105	96	32	16	1 + 2 plasmids	1 + 3 plasmids
Mean contig length	64,056	113,291	43,937	46,393	136,123	274,265	N/A	N/A
Scaffolds	4	14	15	10	4	8	N/A	N/A
Mean sequence gap length	297	246	692	98	285	166	N/A	N/A
Genome size (bp)	4,419,871	4,644,913	4,613,339	4,453,746	4,355,922	4,388,239	4,530,777	4,599,092
CDS	4,041	4,172	4,123	4,077	3,973	3,977	4,211	4,296
G + C%	54.99	56.16	56.84	56.87	56.17	55.79	56.7	57.2
Coverage of contigs	15.33	20.28	13.8	14.63	17.36	25.38	N/A	N/A
tRNAs	79	85	92	86	81	89	82	86
rRNA operons	7	7	7	7	7	7	7	7

^a^*C. sakazakii* strain BAA-894 and *C. turicensis* strain z3032 were previously sequenced and are considered to be closed reference genomes [20,22].

### Whole genome phylogeny and *Cronobacter* taxonomy

**Figure 1:**
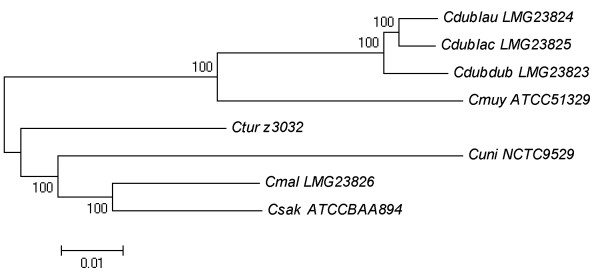
**Evolutionary relationships of eight *Cronobacter *genomes.** Neighbor-Joining phylogenetic tree based on 574,352 bp alignment of homologous sequences was computed using the Maximum Composite Likelihood method for nucleotide substitution. The bootstrap supports, as percentage, are shown next to the branches. The scale bar represents 0.01 base substitutions per site.

To confirm the taxonomic standing of *Cronobacter genus novum* and species [1], genome scale analyses based on nucleotide sequence were performed. Average nucleotide identity (ANI) has emerged as one of the predominant genomics alternatives to DNA-DNA hybridization. The pairwise ANI values between all *Cronobacter* genomes used in this study support the current proposed species and subspecies classification of *Cronobacter* (Table 2). Although pairwise ANI values provide a benchmark of divergence (or similarity) between two genomes, evolutionary relationships between more than two genomes cannot be inferred from this analysis. Therefore, a genome scale phylogenetic analysis was performed on all eight *Cronobacter* genomes (Figure 1). This analysis reveals that extant *Cronobacter* species have diverged into two clusters, Cdub-Cmuy and Csak-Cmal-Cuni-Ctur, from the most recent shared ancestral species. The Cdub-Cmuy clade evolved as a monophyletic clade for much of its evolutionary history before diverging into two species. However, we expect the reconstruction of this evolutionary history to change significantly as new species that fall within this clade are identified; for example, *C*. *condimenti*[5]. In contrast, the other four species evolved as four distinct monophyletic lineages. The phylogenetic tree is, again, in good agreement with the current proposed taxonomy of *Cronobacter*[1], as well as recent proposed *Cronobacter* phylogenies derived from MLST [24] and sequence of the *rpoB* gene [25,26], although more resolving than *rpoB* sequence for Csak-Cmal [27], and *rpoA*[28].

**Table 2:** **Average nucleotide identity (ANI) values for eight *Cronobacter *genomes**

**ANI**	**BAA-894**	**LMG 23825**	**LMG 23823**	**NCTC 9529**	**51329**	**LMG 23826**	**LMG 23824**	**z3032**
*Csak* ATCC BAA-894	---	88.59	88.43	93.28	88.57	94.61	88.57	91.85
*Cdublac* LMG 23825	88.57	---	**97.24**	89.21	91.94	88.77	**98.36**	88.81
*Cdubdub* LMG 23823	88.44	**97.22**^**a**^	---	88.97	91.65	88.54	**97.29**	88.55
*Cuni* NCTC 9529	93.36	89.33	89.04	---	89.16	93.87	89.33	93.71
*Cmuy* ATCC 51329	88.5	92	91.67	89.21	---	88.6	92.03	88.77
*Cmal* LMG 23826	94.59	88.76	88.46	93.77	88.57	---	88.72	92.13
*Cdublau* LMG 23824	88.6	**98.27**	**97.29**	89.26	91.96	88.77	---	88.79
*Ctur* z3032	91.88	88.82	88.54	93.64	88.77	92.1	88.82	---

^a^Numbers in bold represent pairwise comparisons that are higher than the current species level threshold, 95.00 for ANI, indicating same species.

### *Cronobacter* core genome

A *Cronobacter* core genome of orthologous, shared genes was determined for the eight strains analyzed in this study. The chromosome of each of the six type strains of *Cronobacter* consists of approximately 4,000 CDS (Table 1). Of this number, we determined that 3,160 CDS comprise the core genome of *Cronobacter* (Additional file 1: Table S1). Previously, Kucerova et al. [20] reported a core genome of 1,899 gene sequences, among five species using microarray hybridization based on the genome of Csak BAA-894; *C*. *universalis* was not included.

Among the genes comprising the core genome of *Cronobacter*, we found several genetic determinants that are experimentally-linked to phenotypic traits, such as reduction of nitrate, utilization of D-mannose, D-mannitol, sucrose, L-arabinose, cellobiose, and D-xylose (Additional file 1: Table S1), which have been previously reported by Iversen et al. [1] as biochemical traits of the genus as determined by phenotypic microarray. Additionally, we found genes, gene clusters or operons responsible for the following characteristics in the *Cronobacter* core genome: utilization of fructoselysine, psicoselysine, isomaltulose (palatinose), N-acetylglucosamine (*nag*), N-acetylmannosamine, and 2-ketogluconate; a carotenoid pigmentation cluster homologous to *Pantoea* spp., type IV pili (TFP), σ chaperone/usher (CU) fimbriae, three γ1 CU fimbriae, enterobactin siderophore gene clusters, ferric hydroxymate transporter, several ferric iron receptors, and two ferrous iron transporters [29], histidine transporter, *yeh* osmoprotectant transporter, zinc transporter, albicidin resistance protein coding gene, macrolide-specific ABC-type efflux pump and several putative multidrug resistance genes, *opp* oligopeptide transporter system, *dpp* dipeptide transporter, *sap* peptide transporter, cellulose biosynthesis genes (*bcs*, *yhj*), *kps* capsule polysaccharide biosynthesis cluster, glucans biosynthesis cluster, dimethyl sulfoxide reductase, alkanesulfonate monoxygenase and transporter, *ytf* putative sugar transporter operon, and hexuronate operon (Additional file 1: Table S1).

### *Cronobacter* spp. pan-genome

**Figure 2:**
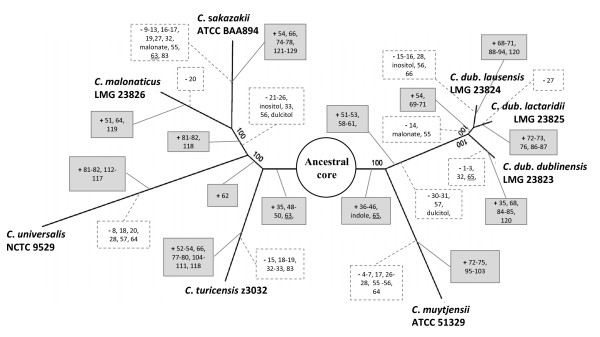
**Gain and loss of putative genomic regions among eight *Cronobacter *genomes.** Genomic regions (see Table 3 and Additional file 4: Table S2 for more details) are shown as present (+, shaded boxes) or absent (−, dashed borders), representing most likely point of gain or loss for each genome or group of genomes. The ancestral core comprises the current eight *Cronobacter* core genome (Additional file 3: Table S1) and GIs 1–34, 55–57, 67, 83. Underlined genomic regions are those whose current species distribution occurred through both insertions and deletions in the evolutionary history of *Cronobacter*.

For the approximately 900 non-core CDS in each of the six *Cronobacter* genomes; i.e., not included in the *Cronobacter* core genome, we determined which chromosomal genes comprised dispensable and unique genomic regions (GR) and which were putative mobile genomic islands and elements. Plasmid genes (approximately 130 CDS per genome, see Additional file 2: Table S2) were not investigated in this study. In total, we identified 84 dispensable genomic regions that were present in two or more *Cronobacter* genomes, and 45 unique genomic regions (Table 3). We overlaid these combined 129 genomic regions onto the genome-scale phylogeny, determined by alignment of orthologous sequence of the core genome (Figure 2).

**Table 3:** **Non-core and unique genomic regions (GRs) identified in eight *Cronobacter *genomes**

***GR#***	***Feature(s) or putative feature(s)***^***a***^	***Cdubdub***	***Cdublac***	***Cdublau***	***Cmuy***	***Ctur***	***Cuni***	***Cmal***	***Csak***	**Shared locus**
1	Acyl reductase-transketolase	-	+	+	+	+	+	+	+	
2	Taurine metabolism	-	+	+	+	+	+	+	+	
3	Environmental persistence capsule, *yih*	-	+	+	+	+	+	+	+	
4	Filamentous hemagglutinin outer membrane protein	+	+	+	-	+	+	+	+	
5	*ybh* transporter, putative antibiotic resistance efflux pump	+	+	+	-	+	+	+	+	
6	γ_4_ fimbriae	+	+	+	-	+	+	+	+	
7	Phospho-alpha-glucosidase (**α-methyl D-glucoside**)	+	+	+	-	+	+	+	+	
8	DMSO, urea (*urt*), biotin utilization, MAR (**4-aminobutyrate**)	+	+	+	+	+	-	+	+	
9	π fimbriae	+	+	+	+	+	+	+	-	
10	Hydrolase	+	+	+	+	+	+	+	-	
11	Transporter and Rhodanese-related sulfurtransferases	+	+	+	+	+	+	+	-	l
12	Lysozyme, virulence regulator	+	+	+	+	+	+	+	-	
13	LPS/core OS 2 gene pair	+	+	+	+	+	+	+	-	
14	MFS transporter, regulator and dehydrogenase	+	-	-	+	+	+	+	+	f
15	Membrane hydrolase	+	+	-	+	-	+	+	+	
16	Large repetitive hemolysin	+	+	-	+	+	+	+	-	
17	Putative ABC transporter and DMT permease	+	+	+	-	+	+	+	-	d
18	Beta-glucosidase	+	+	+	+	-	-	+	+	
19	Hypothetical proteins	+	+	+	+	-	+	+	-	
20	Fatty acid desaturase	+	+	+	+	+	-	-	+	
21	OM autotransporter barrel	+	+	+	+	+	+	-	-	a
22	NO reductase	+	+	+	+	+	+	-	-	
23	Oxidoreductase	+	+	+	+	+	+	-	-	
24	Spermidine-preferential uptake system	+	+	+	+	+	+	-	-	
25	Glucuronyl hydrolase, sucrose permease	+	+	+	+	+	+	-	-	
26	Hypothetical proteins	+	+	+	-	+	+	-	-	
27	Hypothetical proteins	+	-	+	-	+	+	+	-	
28	CRISPR	+	+	-	-	+	-	+	+	
29	**Inositol**	+	+	-	+	+	+	-	-	
30	Non-heme chloroperoxidase, MxcK	-	-	-	+	+	+	+	+	g
31	Acetyltransferase	-	-	-	+	+	+	+	+	
32	Hypothetical proteins	-	+	+	+	-	+	+	-	
33	Putative membrane protein and regulator	+	+	+	+	-	+	-	-	h
34	**Malonate**	+	-	-	+	+	+	+	-	
35	Formate dehydrogenase	+	-	-	-	+	+	+	+	
36	Phenolic sulfur ester	+	+	+	+	-	-	-	-	
37	Deoxyguanosinetriphosphate triphosphohydrolase	+	+	+	+	-	-	-	-	
38	Ferrichrome iron receptor	+	+	+	+	-	-	-	-	
39	Putative membrane and hypothetical proteins	+	+	+	+	-	-	-	-	i
40	Arylsulfatase	+	+	+	+	-	-	-	-	j
41	Redundant methionine transporter	+	+	+	+	-	-	-	-	
42	Ethanolamine permease and deaminase	+	+	+	+	-	-	-	-	
43	Ferrichrome-iron receptor	+	+	+	+	-	-	-	-	
44	Hypothetical proteins	+	+	+	+	-	-	-	-	
45	Ornithine monooxygenase, BtrH	+	+	+	+	-	-	-	-	
46	Transmembrane tRNA/peptide synthetase	+	+	+	+	-	-	-	-	
47	**Indole**	+	+	+	+	-	-	-	-	
48	Putative LPS modification operon	-	-	-	-	+	+	+	+	i
49	Toxin-antitoxin pair	-	-	-	-	+	+	+	+	
50	Exported protein	-	-	-	-	+	+	+	+	
51	CynX	+	+	+	-	-	-	+	-	
52	γ_4_ fimbriae	+	+	+	-	+	-	-	-	l
53	Acyl enzymes	+	+	+	-	+	-	-	-	
54	Membrane protein	-	+	+	-	+	-	-	+	
55	Curli	+	-	-	-	+	+	+	-	
56	Methyl-accepting chemotaxis and/or hypothetical protein	+	+	-	-	+	+	-	-	n
57	Macrophage infectivity potentiator-related protein and regulator	-	-	-	+	+	-	+	+	
58	Putative monooxygenase and transcriptional regulators	+	+	+	-	-	-	-	-	d
59	Oligopeptide transport system	+	+	+	-	-	-	-	-	
60	Hypothetical proteins	+	+	+	-	-	-	-	-	
61	Methyl-accepting chemotaxis and/or hypothetical proteins	+	+	+	-	-	-	-	-	
62	Beta-lactamase	-	-	-	-	-	+	+	+	j
63	Peptide chain release factor	-	-	-	-	+	+	+	-	
64	Chemotaxis	-	-	-	+	-	+	+	-	e
65	Helicase	-	+	+	+	-	-	-	-	
66	Hypothetical proteins	-	-	+	-	-	+	-	+	
67	**Dulcitol**	-	-	-	+	+	+	-	-	
68	Short-chain dehydrogenase and regulator	+	-	+	-	-	-	-	-	d
69	Type I RMS	-	+	+	-	-	-	-	-	c
70	Pyroxidine/pyroxidal metabolism	-	+	+	-	-	-	-	-	e
71	*qseBC*, *ompV*	-	+	+	-	-	-	-	-	f
72	Maltose phosphate PTS and glucosidase	-	+	-	+	-	-	-	-	
73	Cellulose degradation	-	+	-	+	-	-	-	-	
74	Transporter and secreted pair	-	-	-	+	-	-	-	+	
75	Hypothetical proteins	-	-	-	+	-	-	-	+	
76	β fimbriae	-	+	-	-	-	-	-	+	
77	Periplasmic or lipo-protein	-	-	-	-	+	-	-	+	
78	α-mannosidase	-	-	-	-	+	-	-	+	
79	Regulator and hypothetical protein	-	-	-	-	+	+	-	-	
80	Short chain dehydrogenase	-	-	-	-	+	+	-	-	
81	Membrane protein	-	-	-	-	-	-	+	+	
82	γ_1_ fimbriae	-	-	-	-	-	-	+	+	
83	(+/−) putative membrane and (+/−) hypothetical proteins; variable	+	+	+	+	-	+	+	-	
84	Arsenic resistance; MFS transporter, regulator and hypothetical proteins	+	-	-	-	-	-	-	-	i
85	Putative monoamine oxidase, carboxymuconolactone decarboxylase and hypothetical proteins	+	-	-	-	-	-	-	-	l
86	Hypothetical proteins	-	+	-	-	-	-	-	-	
87	Putative monoamine oxidase, carboxymuconolactone decarboxylase and hypotheticals; homologous to GR85	-	+	-	-	-	-	-	-	i
88	Putative sulfatase and hypothetical proteins	-	-	+	-	-	-	-	-	a
89	LysE Superfamily exporter	-	-	+	-	-	-	-	-	d
90	Hypothetical proteins	-	-	+	-	-	-	-	-	
91	Radical SAM and ABC ATPase proteins	-	-	+	-	-	-	-	-	
92	Mannanase	-	-	+	-	-	-	-	-	g
93	Putative reductases and hypothetical proteins	-	-	+	-	-	-	-	-	i
94	Membrane protein	-	-	+	-	-	-	-	-	k
95	D-glucarate permease, transketolase, homologous to GR117	-	-	-	+	-	-	-	-	c
96	Type I RMS	-	-	-	+	-	-	-	-	d
97	Modulator of drug activity B (*mdaB*), MFS transporter and regulator	-	-	-	+	-	-	-	-	n
98	Hypothetical proteins	-	-	-	+	-	-	-	-	
99	Hypothetical proteins	-	-	-	+	-	-	-	-	h
100	Hypothetical proteins	-	-	-	+	-	-	-	-	
101	FHA	-	-	-	+	-	-	-	-	
102	L-rhamnose ABC transporter	-	-	-	+	-	-	-	-	
103	Urea carboxylase	-	-	-	+	-	-	-	-	
104	Heteropolysaccharide degradation	-	-	-	-	+	-	-	-	b
105	*qseBC*, *ompV*	-	-	-	-	+	-	-	-	d
106	Sigma factor, catalase	-	-	-	-	+	-	-	-	k
107	Beta-glucosidase, cellobiose	-	-	-	-	+	-	-	-	
108	Short chain dehydrogenase	-	-	-	-	+	-	-	-	
109	Paralogous enzyme island	-	-	-	-	+	-	-	-	
110	DND operon	-	-	-	-	+	-	-	-	
111	Xanthosine utilization	-	-	-	-	+	-	-	-	
112	κ fimbriae	-	-	-	-	-	+	-	-	
113	Putative epoxide hydrolase	-	-	-	-	-	+	-	-	b
114	Cupin superfamily homologues	-	-	-	-	-	+	-	-	d
115	Hypothetical proteins	-	-	-	-	-	+	-	-	
116	Hypothetical proteins	-	-	-	-	-	+	-	-	i
117	D-glucarate permease, transketolase	-	-	-	-	-	+	-	-	
118	Large exoproteins involved in heme utilization or adhesion	-	-	-	-	+	-	+	+	
119	MipA homologue and signal transduction pair	-	-	-	-	-	-	+	-	i
120	Hemolysin, activator and secretion	+	-	+	-	-	-	-	-	
121	Membrane spanning and hypothetical proteins	-	-	-	-	-	-	-	+	a
122	Hypothetical proteins	-	-	-	-	-	-	-	+	
123	Csak invasin locus	-	-	-	-	-	-	-	+	
124	Unique transporter	-	-	-	-	-	-	-	+	e
125	Nucleoside-sugar efflux	-	-	-	-	-	-	-	+	
126	γ_1_ fimbriae	-	-	-	-	-	-	-	+	
127	*nanC*, sugar isomerase, hydrolase	-	-	-	-	-	-	-	+	
128	Paralogous enzyme island	-	-	-	-	-	-	-	+	
129	Sialic acid	-	-	-	-	-	-	-	+	

^a^Feature(s), or putative feature(s), were assigned based on BLASTp homology of encoded ORFs to known proteins. Shared chromosomal loci of genomic regions are indicated by matching letters. Features in bold are biochemical traits, used previously to biotype *Cronobacter* isolates.

In addition to the extant *Cronobacter* spp. core genome described earlier (Additional file 1: Table 1), a number of dispensable or accessory genomic regions, 39 in all, were identified as being a likely component of the last common ancestor core genome (Figure 2). These included metabolic operons for the utilization of taurine, inositol, malonate, and dulcitol (galactitol); two chaperone-usher fimbriae operons and curli biosynthetic genes; a CRISPR element; an alpha- and beta-glucosidase; genes encoding the *yih* environmental persistence capsule described in *Salmonella* species; two large repetitive protein/hemolysin clusters; spermidine uptake system; nitric oxide reductase system; a 58.4-kb genomic region missing in Cuni NCTC9529 containing gene clusters for the reduction of dimethyl sulfoxide, uptake of biotin and urea (*urt*), and a multiple antibiotic resistance cluster; as well as several genomic regions containing largely hypothetical protein encoding genes (Figure 2, Table 3).

We also identified 45 non-core dispensable genomic regions, which were acquired by two or more species of *Cronobacter* after diverging from their most recent common ancestor. This subset of genomic regions included 12 that were present in the Cdub-Cmuy cluster and included two ferrichrome iron receptors, *fcT* and *fcuA* orthologues [29]; methionine and ethanolamine utilization gene clusters; and tryptophanase (indole). Additional non-core genomic regions included a β class (GR76, Csak and Cdublac), a γ1 class (GR82, Cmal and Csak) and a γ4 class chaperone-usher fimbriae (GR52, Cdub and Ctur); an oligopeptide transport system (GR59, Cdub); pyridoxine metabolism cluster (GR70, Cdublac and Cdublau); maltose uptake system (GR72, Cdublac and Cmuy); cellulose degradation operon (GR73, Cdublac and Cmuy); and an α-mannosidase (GR78, Csak and Ctur).

Each *Cronobacter* genome contained a number of unique genomic regions; however, the vast majority of these contained genes encoded hypothetical proteins. Notable exceptions to this trend were genomic regions encoding for mannan degradation (GR92, Cdublau); L-rhamnose ABC transporter (GR102, Cmuy); urea decomposition (GR103, Cmuy); *dnd* (degradation during electrophoresis), DNA sulfur modification system, (GR110 Ctur); heteropolysaccharide degradation (GR104, Ctur); putative epoxide hydrolase gene cluster (GR113, Cuni); κ class chaperone-usher fimbriae (GR112, Cuni); invasin locus (GR123, Csak); γ1 class chaperone-usher fimbriae (GR126, Csak); and sialic acid utilization (GR127 and 129, Csak) (Table 3).

A number of chromosomal loci were found to harbor multiple genomic islands (Table 3, shared locus); for example, GR 33 and 99 in Cmuy ATCC 51329 (Table 3). Additionally, there were two insertion sites; namely, tRNA-Pro-GGG and the TCA cycle isocitrate dehydrogenase gene, *icdA*, where several small genomic regions were inserted in a multiple cassette fashion for several genomes, indicating insertion loci of genomic plasticity (Table 3, Table 4).

**Table 4:** **Putative mobilome of *Cronobacter *genomes**

***Putative mobile element***	***Insertion site***	***Other elements at this chromosomal locus***
T6SS1 (Csak)	tRNA-Phe-GAA	
T6SS2 (Csak, Cmal, Cuni, Cdublau, Ctur)	tRNA-Phe-GAA	GR 21, 88, 121
Transposon-Phage (Cuni)	tRNA-Met-CAT	GR 122 (Csak)
Prophage (Cdublac, Ctur); small integron (Cmal)	tRNA-gly-CCC	GR 112 (Cuni)
Prophage (Cdublac, Cmal, Ctur); tandem prophages (Cdubdub); Prophage-transposon (Cdublau); transposon (Cuni)	putative serine chemoreceptor protein gene	GR 16
Prophage (Csak)	large repetitive protein gene	
IS (Cmuy)	*yfgG*	GR 104, 113
Prophage (Cdubdub, Cmal); transposon (Ctur)	tRNA-Arg-CCT	GR 69, 95
Prophage (Cdubdub, Csak)	tRNA-Pro-GGG	GR 17, 58, 68, 89, 96, 105, 114, 123
TE (Cmal)	hydroxyethylthiazole kinase gene	
O-antigen region	*galF-gnd*	
IS (Cmuy)	tRNA-Asn-GTT	GR 27
T6SS3 (missing in Cmuy)	tRNA-Asn-GTT	
Prophage (Cdublau); T6SS4 with transposon interruption (Cdublau), or at end (Cdublac)	tRNA-Asn-GTT	
Prophage (Cmal)	*fliE*	
ICE (Cmuy)	*msdB-pykA* intergenic	
Prophage (Cdubdub)	SraC/RyeA RNA	GR81
Prophage (Cmal); TE (Ctur)	*ycgN*	
Transposon (Cdublau)	interruption GR 59	
Prophage (Cdublau, Cmuy, Csak)	*ycjR-ycjS*	
**Tellurium resistance transposon** (Csak)	interruption GR 8	
Transposon (Cuni)	GR 30	GR 92
Transposon (Cdubdub)	*uvdE*	
IS600 (Cdub); Prophage (Cdublau)	*ydcW* intergenic	GR 31 (variable)
Prophage (Cuni)	*phnO* intergenic	
T6SS5 (highly variable, missing in Cdublac, Cmuy)	tRNA-Val-GAC	GR 33, 99
**Copper-resistance transposon** (Cuni (+ABC transporter), Cmuy); Prophage (Cmuy, Ctur)	*icdA*	GR 39, 48, 84, 87, 93, 116, 119
Prophage (Csak)	*ycdX*	
Prophage (Cdublau)	*mntR-ybiP* intergenic	
Prophages (Cuni, Csak, Ctur); T6SS6 (Cdub, Ctur, Csak - truncated)	tRNA-Arg-TCT	GR 11, 52, 85
Prophage (Csak); Transposon (Cmal), TE (Cdublau)	tRNA-Thr-CGT	
TE (Cmal)	*pilB*	
Prophage (Cdublac, Cmal); TE (Cdublau, Cdubdub, Cuni); Transposon (Cdublau)	tRNA-Leu-CAA	GR 110
ICE (Cuni)	large repetitive protein gene	
**T6SS7**-repeat region (all, variable and conserved genes)	*tpiA* intergenic	
Small integron (Csak)	*yicC-yicG*	
Core OS variable region	*kdtB-kbl*	
Prophage (Cmuy); TE (Cdubdub, Cuni)	tRNA-SeC(p)-TCA	
**Copper and silver-resistance transposon** (Csak)	*yhiN-puuA*	

### *Cronobacter* spp. mobilome

**Figure 3:**
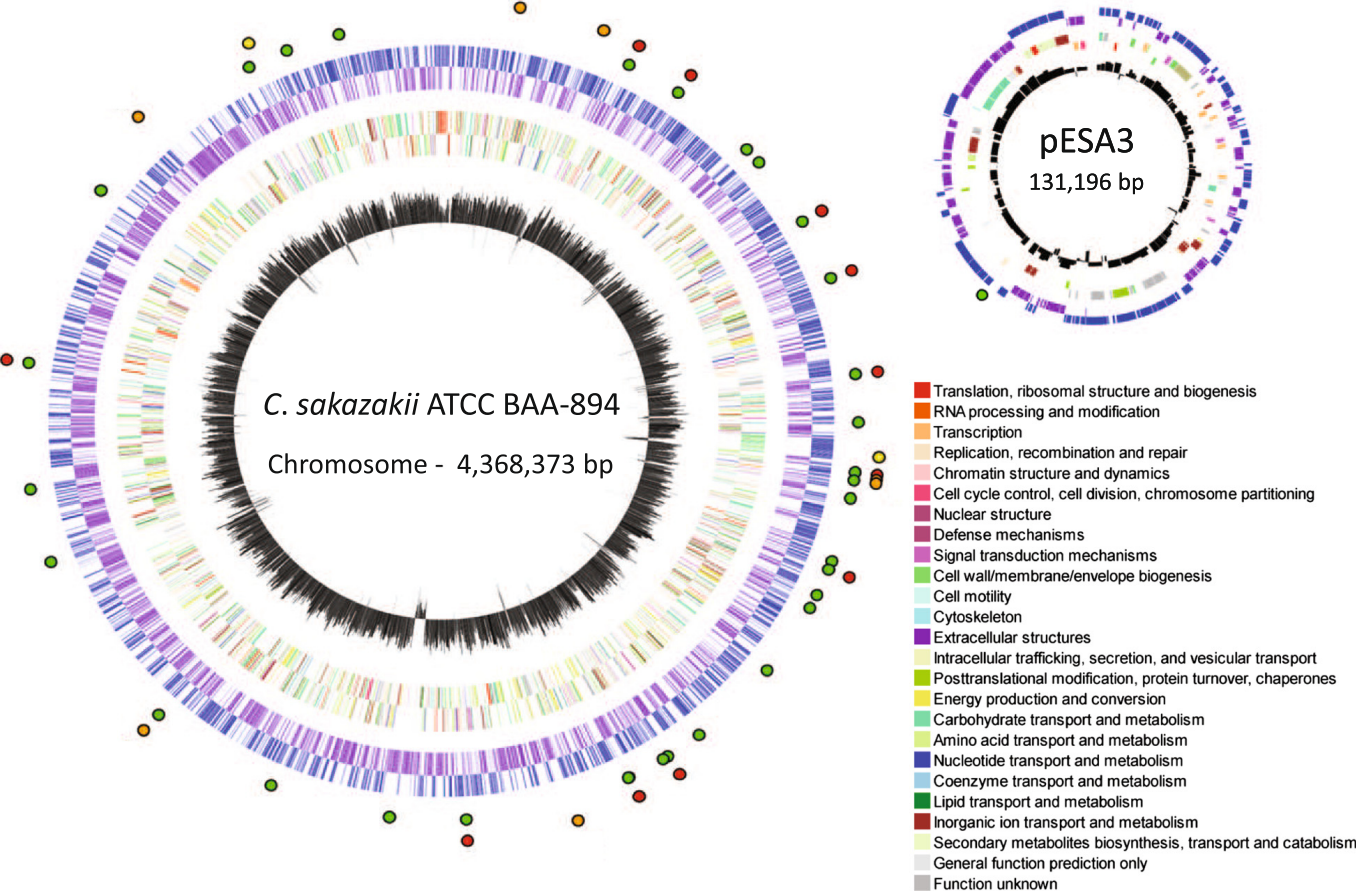
**Mobilome of *Cronobacter *spp.** Putative mobilome elements from eight *Cronobacter* genomes were mapped on *C. sakazakii* ATCC BAA-894 chromosome, using the Microbial Genome Viewer [31]. Symbols on outside ring represent insertion sites of putative mobile genetic elements (Table 4, with mobile elements plotted clock-wise, from the top) – prophages, transposons and insertion sequences, and integrative-conjugative element-like elements (green circles), variable type six secretion system clusters (orange circles), genomic regions at insertion sites (red circles), and LPS (O-antigen and core OS) regions (yellow circles). Blue ring represents genes on the + strand and purple ring represents genes on the – strand. Middle rings represent COGs on + (outer) and – (inner) strands (COG functional category legend provided in lower right of figure). Innermost circle represent % G + C.

Not surprisingly, each *Cronobacter* genome contained multiple prophage or prophage-like elements (Table 4, Figure 3). In several cases, prophages of different *Cronobacter* genomes were inserted at the same chromosomal locus (Table 4, Figure 3). Indeed, phylogenetic analysis of the integrase gene of all prophages revealed a trend of several clusters in which the chromosomal insertion site was shared among all members of a phylogenetic cluster (Additional file 3: Figure S1). Interestingly, the genomes of *C*. *universalis* NCTC 9529 and *C*. *muytjensii* ATCC 51329 harbor a 57 kb integrative conjugative element (ICE), which is 99% identical between strains (Table 4). This mobile genetic element is most closely related to the ICE-KKS family of integrating and conjugative elements, found in β- and γ- proteobacteria and represented by ICE_KKS102_*4677*, which carries polychlorinated biphenyl degradation genes [30]. For the *Cronobacter* ICE, in addition to the conjugal transfer, integrase, and replication/partition genes, there is a 21 kb internal segment which carries unique genes among this class of ICEs. Most of the genes were annotated as encoding hypothetical proteins, so it is unclear what phenotypic trait or characteristic(s) are encoded in this region.

Additionally, each genome contained a number of transposons (Table 4). Phylogenetic analysis of the transposases clustered the genes together based on multiple copies of the same transposon found in each genome, but not with regard to insertion site (Additional file 4: Figure S2). Most of the transposons carried very few additional genes or hypothetical protein encoding genes. There were three noticeable exceptions, a transposon carrying the tellurium resistance island found in the genome of Csak BAA-894, a transposon carrying a copper resistance island found in the genomes of Cuni NCTC 9529 and Cmuy ATCC 51329, inserted near the isocitrate dehydrogenase gene, *icdA*, and a transposon inserted in the *yhiN* - *puuA* intergenic region of Csak BAA-894, carrying copper and silver resistance genes (Table 4, Additional file 2: Table S2).

We also observed a number of type six secretion system (T6SS) gene clusters in the eight *Cronobacter* genomes (Table 4, Figure 3, Additional file 2: Table S2). All genomes contained a very large T6SS cluster as a component of the *Cronobacter* core genome, which is flanked by a highly variable region of different sizes in each genome containing several hypothetical protein CDS as well as those encoding numerous homologues of *vgrG*, Rhs-family and YD-repeat proteins (Table 4, T6SS7; Additional file 2: Table S2). Additionally, seven of the eight genomes contain four to six accessory T6SS clusters; Cmuy ATCC 51329 does not contain any additional T6SS clusters. The gene content of each cluster is variable between clusters present at different chromosomal loci, but largely conserved among clusters located at the same chromosomal locus. Previously, we reported the presence of a T6SS cluster on a repFIB plasmid in strains of *C*. *sakazakii*[23].

Not surprisingly, many of the mobile genetic elements, such as lysogenic prophages, in *Cronobacter* genomes are inserted at tRNA loci (Table 4). We also found T6SS gene clusters and some genomic regions inserted at tRNA sites. And as with genomic regions, we observed cassette-like insertion of multiple types of genetic elements at single sites (Table 4).

## Discussion

Like many bacterial genera, the taxonomy of *Cronobacter* has evolved and expanded as more sensitive molecular- and sequence-based tools have developed. In this study, we performed two genome-scale sequence analyses to discern the taxonomic relationships of extant *Cronobacter* species, namely ANI and genome-scale alignment and phylogenetic reconstruction using syntenic, orthologous chromosomal sequence. The taxonomic reclassification by Iversen et al. [1], which relied on both DNA studies and on results from biochemical tests, was confirmed by both analyses. We found that the ANI results from this study were more meaningful in discerning relationships between pairs of *Cronobacter* species that are more distantly related as compared to DNA-DNA hybridization [1]. This is most likely a reflection of the differences in the range of meaningful values for each analysis.

We were able to confirm the presence or absence of eight of the genetic determinants of the biochemical characteristics used previously for *Cronobacter* biotyping (Table 3); namely, indole (tryptophanase), dulcitol (galactitol), malonate, *myo*-inositol, and two genomic regions that are likely responsible for utilization of 4-aminobutyrate and production of α-methyl glucoside (Farmer biotype 15 [2]), as well as those biotyping traits contained in the core genome of these eight strains, utilization of palatinose and putrescine (Additional file 1: Table S1). The distribution of these operons and genes were in complete agreement with the biochemical results and species description reported by Iversen et al. [1]. Inositol fermentation has recently been proposed as a marker of pathogenicity for *Cronobacter*, based on the presence of the inositol monophosphatase gene (*suhB*) in pathogenic strains [32]. In this study, we found that this gene, which is seemingly ubiquitous and highly conserved among the *Enterobacteriaceae*, is a component of the *Cronobacter* core genome (Additional file 1: Table S1). Additionally, we found that the inositol utilization operon (GR29, Table 3, Additional file 2: Table S2) was present, and functional [1], in the genomes of strains isolated from the environment (water sources), Cuni NCTC 9529^T^, Cdubdub LMG 23823^T^, Cdublac LMG 23825^T^, and absent in the genomes of pathogenic strains, Cmal LMG 23826^T^ and Csak BAA-894.

Using comparative genomics, we were able to define the syntenic *Cronobacter* core genome for the eight species genomes analyzed in this study, which is approximately 77% of the total protein coding sequences, on average, per genome. This value is considerably higher as compared to the core genome content of other genera. In fact, the core genus genome size of *Cronobacter* is comparable to the core genome size of certain bacterial species [33,34], and considerably larger than that of the related *E*. *coli*[35]. This is a reflection of the phylogenetic closeness of this genus, as shown in the ANI results (Table 2), and indicative of a more “closed” *Cronobacter* genome.

The core genome size is considerably higher than that reported by Kucerova et al. [20], 1,899 genes, which incorporated four of the six strains used in this study. This discrepancy is best explained by the divergent evolution of the genus to form two distinct clades (Figure 1). This divergent evolution would undoubtedly have a significant impact on the efficiency of hybridization of probes designed from the sequence of Csak BAA-894 to DNA from strains of Cdub and Cmuy, resulting in the smaller reported core genome size [20]. With regard to the genomic regions revealed by the comparative genomic hybridization analysis of Csak BAA-894, reported by Kucerova et al. [20], we classified 12 of the 15 reported genomic regions as part of the *Cronobacter* mobilome. They included T6SS clusters, GR1, 2 (ESA_0292-00302), 9, and 15 (repFIB plasmid-associated T6SS); putative prophages, GR3, 4, 6, 10, 11, 12; transposons, GR7, 14; and O-antigen gene clusters, GR5 (Additional file 2: Table S2, from [20]).

The comparably large core genome size and overall high sequence identity within the genus support the hypothesis that these two clades have evolved as a result of sympatric speciation; however, the divergence of the two clades indicates that they are under different evolutionary pressures, to some degree. In addition to the divergent evolution of shared genome content, several non-core genomic regions were found to be either present (or absent) in one of the two species-complex clades. For example, the Cdub-Cmuy clade has acquired 13 genomic regions that are not present in the Csak-Cmal-Cuni-Ctur clade (Figure 2).

An examination of the dispensable gene content of each species provides clues as to differences in environmental niches and pathogenicity, such as virulence potential. Among putative virulence-related properties, the presence and diversity of appendages within the *Cronobacter* genus is intriguing. Many causative agents of meningitis, such as *Neisseria meningitidis*, *Haemophilus influenzae*, and *Pseudomonas aeruginosa* encode for type IV pili (TFP), necessary to colonize in the face of shear forces of blood flow, associated with the capillary beds of the blood–brain barrier. The *Cronobacter* core genome also contains the genes that encode a TFP. Bioinformatically, the presence of a TFP, as opposed to a T2SS, was hypothesized due to the presence of the TFP-unique gene, *pilT*, and the genetic organization of the *pilQ* loci, similar to the TFP of *P*. *aeruginosa*. Several chaperone-usher fimbriae were also present in the core genome. Additionally, seven other chaperone-usher fimbriae were differentially distributed among the eight genomes analyzed in this study (Table 3), including a P pilus homologue (GR9, missing in Csak BAA-894), which is a prominent virulence factor of uropathogenic *E*. *coli*, also a causative agent of neonatal meningitis. Csak BAA-894 and Cmal LMG 23826^T^ harbor a unique type I fimbriae (GR82), which is absent in the other genomes, and Csak BAA-894 also harbors a β class CU fimbriae (GR76), shared with Cdublac LMG 23825^T^, and a second, unique type I fimbriae (GR126, which corresponds to GR8 of Kucerova et al. [20]). Of interest is the finding that Cmuy ATCC 51329^T^ possesses only one additional fimbriae operon, in addition to those encoded in the genus core genome. Some genomes also harbored curli biosynthesis genes, homologous to curli of *E*. *coli* and tafi fimbriae of *Salmonella*. Although implicated directly in cell-cell contact and biofilm formation, these organelles likely contribute to the colonization of *Cronobacter*, after initial cell attachment has taken place. We hypothesize that this operon was a component of the ancestral core, and has been lost in all strains of *C*. *sakazakii* and *C*. *muytjensii* (data not shown).

In addition to appendages potentially involved in adhesion, several type V(a), or autotransporter, secretion loci are present in the genomes of the *Cronobacter* analyzed in this study, which are annotated as hemolysin, adhesin, outer membrane autotransporter barrel, filamentous hemagglutinin, large exoproteins, etc. They are found as accessory genomic regions (GRs 4, 16, 21, 50, 101, 123), and present as single genes or pairs of genes in the core genome (Additional file 2: Table S2). Of particular interest is GR123, found exclusively in Csak BAA-894, and GR118, found in the three routinely isolated pathogenic species, Ctur, Cmal, and Csak. GR123 contains two putative invasins and an *eae* homologue and may constitute a pathogenicity island. This region was found to be present in the genomes of three neonatal intensive care unit (NICU) outbreak strains (including Csak BAA-894) and absent in the *C*. *sakazakii* type strain, ATCC 29544, which was isolated from a child’s throat (defined as a component of cluster 3, Additional file 1: Table S1, from [20]).

Also interesting is the presence of two genomic regions (GRs 127 and 129) involved in the utilization of sialic acid in the genome of Csak BAA-894. Sialic acid is a generic term for a family of derivatives of the nine carbon sugar acid, neuraminic acid, which are found at surface-exposed end positions of eukaryotic, primarily animal, tissues. Many pathogens have evolved to either coat their surfaces with sialic acid derivatives, in order to evade the innate immune response, or to use this biopolymer as a nutrient source [36]. In addition to Csak BAA-894, we found that 55 out of 57 strains of *C*. *sakazakii* are able to utilize N-acetyl-neuraminic acid, a derivative of sialic acid (by Biolog PM Microarray, data not shown). Conversely, no other *Cronobacter* strains were able to utilize this substrate, except four of six *C*. *turicensis* strains (data not shown).

It has been hypothesized that the environmental niche of *Cronobacter* is as a plant commensal [37]. Accordingly, we found several genomic features, both in the *Cronobacter* core- and pan-genome, which would be beneficial for an organism to possess in this habitat. For example, the *Cronobacter* core genome contains the maltose transporter operon, *malGFE- malKlamBmalM*, repressor, *malT*, and α-glucosidases that can hydrolzye maltose to two glucose molecules. Maltose is primarily restricted to plants, particularly seed tissues. An operon for the transport and hydrolysis of isomaltulose is also present in the core genome of *Cronobacter*, in agreement with the taxonomic description of Iversen et al. [1], and previously reported by Lehner et al. [38]. Isomaltulose (palatinose), also used in the original *Cronobacter* biotyping scheme, is a disaccharide of glucose and fructose and a component of honey and sugar cane. Additionally, we found the following characteristics in the core *Cronobacter* core genome: utilization of arabinogalactan, a major component of plant gums; transport and utilization of xylose, a precursor to hemicellulose; galacturonate, the principal component of pectin; albicidin, a phytotoxin of *Xanthomonas* spp., resistance; β-carotene pigmentation, and several α and β glucosidases.

It is of interest to find that an albicidin resistance protein coding gene was found as a core genome component. Albicidin is a bacteriocin-like molecule that degrades DNA gyrase, both of bacterial and chloroplast origins [39]. Speculatively, *Cronobacter* possessing a gene promoting resistance to the action of albicidin adds further evidence for a plant-associated evolutionary history, as well as, the impartation of a competitive edge to *Cronobacter* survival in a mixed organism environment where competition is controlled through the action of bacteriocin expression. In addition to these conserved features, several other genomic regions and operons were found that have putative functions for plant association, or homologies to proteins from plant commensals. These include GR95/117 of Cmuy ATCC 51329 and Cuni NCTC 9529; GR70, metabolism of pyroxidine/pyroxidal (vitamin B_6_), of which green plants and grains and nuts contain high amounts; GR72, maltose derivative metabolism; GR73, galactose (glycoside-pentoside-hexuronide) homologue permease (possible role in cellulose degradation); GR92, mannanase; GR102, L-rhamnose ABC transporter; and GR107 of Ctur z3032.

Several inherent properties of *Cronobacter* have been proposed as mechanisms that aid the bacteria in survival and persistence in dried foods, such as PIF, food powders, and spices. Chief among these have been enhanced heat resistance, as compared to other enterics and contaminating microorganisms. However, most studies have reported variable results in terms of heat resistance at the strain level, and cross-tolerance to other environmental stressors, such as pH and water activity. One consistent finding is an unusually high resistance to dry stress [17]. Accordingly, we found several genomic determinants, which would be beneficial in a dry or low water activity environment, including cellulose biosynthesis (*bcs* and *yhj*) operons, colanic acid EPS, capsular biosynthesis operon (*kps*), an environmental persistence capsule (*yih*, GR3), and curli (GR55). Recently, it has been reported that the synergistic expression of the *yih* operon encoded capsule, cellulose and curli or tafi provides resistance to desiccation stress in *Salmonella*[40,41]. We hypothesize that the same genetic determinants, combined with other capsular and EPS operons, likely play a similar role in the environmental persistence and desiccation resistance in *Cronobacter*. Although not all *Cronobacter* produce curli, those species possess several other fimbriae which could substitute in this adhesin and/or scaffolding role. In addition to this extracellular matrix, we also found two operons, present in all *Cronobacter* genomes, that encode transporters involved in osmoprotection, *yeh* and *bet* operons, with homology to plant commensals and pathogens, such as *Burkholderia* and *Erwinia* species.

## Conclusions

In conclusion, we found a large core genome among representative type species strains of the genus *Cronobacter*, which encodes factors for enhanced environmental persistence and plant commensalism, as well as numerous appendages that may aid in attachment and colonization, advantageous both in the environment and in the host. Conversely, we found that the genus has diverged in a bidirectional manner. The Cdub-Cmuy clade has evolved to be more adapted for primarily an environmental and plant association niche, while the other clade, particularly Csak and Cmal, has evolved and acquired accessory genes that have enhanced its virulence capacity, and host species adaption, and promoted pathogenicity. Clearly, *in silico* analysis of strain level differences, coupled with experimental studies, will reveal which of these factors are most important for environmental, plant, and human pathogenic lifestyles of this group of organisms. This study establishes a powerful platform for further functional genomics research of this diverse group; an important prerequisite towards future development of countermeasures against this foodborne pathogen.

## Methods

### Bacterial strains

*Cronobacter dublinensis* subsp. *dublinensis* LMG 23823, *C*. *dublinensis* subsp. *lausannensis* LMG 23824, *C. dublinensis* subsp. *lactaridi* LMG 23825, *C. malonaticus* LMG 23826, and *C. universalis* NCTC 9529 were acquired from Dr. Carol Iversen; *C. muytjensii* ATCC 51329 was acquired from the American Type Culture Collection (ATCC; Manassas, VA).

### DNA sequencing and assembly

Genomic DNA was fragmented to an average size of 3 kb using a Covaris (Woburn, MA) E210 focused-ultrasonicator. Then, Multiplex Identifier (MID)-tagged, paired-end libraries were prepared using a modified version of the 454 Life Sciences (Roche, Branford, CT) protocol. The procedure has been adapted to 96-well plate format using automated pipetting robots, and includes QC steps and AMPure (Beckman-Coulter (Agencourt), Indianapolis, IN) bead-based DNA purifications between enzymatic reactions as described by Fisher et al. [42]. Libraries were pooled and sequenced using the Roche 454 FLX pyrosequencer to an average depth of 15× coverage. Raw sequence data were processed using manufacturer’s software and quality filtering algorithms. Resulting demultiplexed data sets were assembled using Celera Assembler v6.1 (http://sourceforge.net/projects/wgs-assembler/).

### Annotation and comparative genomics

Genomic contigs were submitted as multiple-sequence FASTA files and annotated using the RAST annotation server [43], to identify RNAs and protein-coding genes. The RAST server is a free web portal (rast.nmpdr.org), provided by the SEED [44], which automatically and rapidly curates both closed and draft genomes using a subsystems approach, as opposed to a tedious gene by gene method. Comparative genomics was performed as previously reported [29,44-47], with some exceptions. In this study, genome to genome comparisons were performed primarily with the SEED viewer [48], which utilizes bi-directional protein-protein BLAST (blastp) sequence comparison of translated ORFs. Because sequencing resulted in draft genomes (Table 1), we used the closed genomes of *C*. *sakazakii* ATCC BAA-894 (BioProject Accession PRJNA12720) and *C*. *turicensis* z3032 (BioProject Accession PRJEA39965) as references to align draft contigs using MUMmer [49]. The syntenic core genome of *Cronobacter* was determined using the SEED viewer sequence based comparative genomics tool. To ensure the most complete and robust syntenic core gene set among the genomes analyzed, the genomes of *C*. *sakazakii* ATCC BAA-894 and *C*. *turicensis* z3032 were uploaded and annotated via the RAST server, and used as reference genomes for comparative genomics. For the draft genomes, genes at the end of a contig or interrupted by contig gaps were reconciled using bi-directional BLASTN analysis, against all other genomes.

Genomic regions (GR), defined as regions which are present in one or more *Cronobacter* genomes and missing in at least one other genome (dispensable), were identified as previously reported [44,45,47]. Most probable insertion and deletions of genomic regions were estimated as done previously [44], using a maximum parsimony approach. Clade-specific, as well as ancestral genome genomic regions were identified by collapsing shared genome genomic regions to the farthest branch point that maintained the most parsimonious outcome. For clarity, only the last common ancestor to all eight *Cronobacter* spp. genomes analyzed in this study is shown in Figure 2. It is assumed that each branch point would produce a hypothetical ancestral genome common to those genomes beyond that branch point. Genomic region identification numbers were assigned such that genomic regions that are unique or shared by an individual *Cronobacter* genome were successively numbered. Mobile genetic elements were identified in a similar fashion as genomic regions. In addition, mobility was determined based on significant identity alignments, using BLASTP, of its member ORFs to phage integrases and structural genes, transposases, and conjugative pili transfer genes, contained in the GenBank NR database.

Average nucleotide identity, by BLAST, (ANI) [50] was computed using the JSpecies package [51]. A genome-scale phylogeny was computed as previously described [44,47,52], with some exceptions. As opposed to using sets of orthologues of individual genes, 16 conserved chromosomal syntenic regions, were first aligned individually using MEGA version 5 [53], and then concatenated. These regions spanned the following gene loci from *C*. *sakazakii* ATCC BAA-894 (GenBank CP000783.1), ESA_00107-00117, 00146–00223, 00399–00443, 00671–00737, 01048–01094, 03220–03278, 03280–03289, 03331–03353, 03358–03402, 03447–03511, 03536–03550, 03554–03599, 03614–03648, 03871–03886, 03982–04015, 04016–04029, and included 591 coding sequences (CDS) and intergenic regions. Phylogenetic reconstruction was performed in MEGA, using the neighbor-joining method. The bootstrap consensus tree shown in Figure 1 was inferred from 1000 replicates. The percentage of replicate trees in which the associated taxa clustered together in the bootstrap test (1000 replicates) are shown next to the branches. The tree is drawn to scale, with branch lengths in the same units as those of the evolutionary distances used to infer the phylogenetic tree, i.e., the number of base substitutions per site, as determined using the Maximum Composite Likelihood method. The original dataset included 600,341 bps. All positions containing gaps and missing data were eliminated. There were a total of 574,352 positions in the final dataset.

### Accession numbers

The Whole Genome Shotgun projects described in this study have been deposited at DDBJ/EMBL/GenBank under the accessions, *C*. *dublinensis* subsp. *dublinensis* LMG 23823, [GenBank: AJKZ00000000]; *C*. *dublinensis* subsp. *lausanensis* LMG 23824, [GenBank: AJKY00000000]; *C*. *dublinensis* subsp. *lactaridii* LMG 23825, [GenBank: AJKX00000000]; *C*. *malonaticus* LMG 23826, [GenBank: AJKV00000000]; *C*. *muytjensii* ATCC 51329, [GenBank: AJKU00000000]; and *C*. *universalis* NCTC 9529, [GenBank: AJKW00000000]. The versions described in this paper are the first, XXXX01000000.

## Competing interest

All of the authors declare that they have no competing interests.

## Authors’ contributions

CJG carried out the comparative genomics analyses and wrote the paper. MLK coordinated DNA sequencing with IGS (Baltimore, MD). All authors helped to draft the manuscript. FP, CI, AL, RS, MLK, GG, SF, KAP, CJG, AAF, KGJ, VS, and BDT conceived of the study and of the concepts involved in the formation of an international *Cronobacter* consortium. All authors participated in the study’s design and in the coordination of efforts by the consortium. All authors read and approved the final manuscript.

## Supplementary Material

Additional file 1: Table S1 Core genome of eight *Cronobacter* spp. Core genome components are shown, using Csak ATCC BAA-894 as a reference genome, to include contig, start, stop, length in bp, and % identity (BLASTn). Genomes were annotated using the RAST server; gene identification numbers (ID) were assigned by RAST. Click here for file

Additional file 2: Table S2 Feature tables of eight *Cronobacter* genomes. Annotated feature tables, generated by RAST, of the eight *Cronobacter* genomes used in this study. Each feature (column E) was assigned as a component of: a genomic region (GR, column A), the mobilome (column B), a plasmid (column C), or the core *Cronobacter* genome (column D); however, not all features could be assigned to one of these classifications. Several feature characteristics are presented. The feature table for each genome is given as a separate worksheet, named according to the particular strain. Click here for file

Additional file 3: Figure S1 Phylogenetic relationship of integrases of eight genomes of *Cronobacter* spp. See Additional file 2: Table S2 for further details of each putative integrase. Notation to the right of each integrase indicates insertion locus. Click here for file

Additional file 4: Figure S2 Phylogenetic relationship of transposases of eight genomes of *Cronobacter* spp. See Additional file 2: Table S2 for further details of each putative transposase. Notation to the right of each transposase indicates insertion locus. Click here for file
